# Online Social Support and Trait Anxiety and Phubbing in Nurses

**DOI:** 10.1192/j.eurpsy.2023.214

**Published:** 2023-07-19

**Authors:** A. Şafak, F. Oflaz

**Affiliations:** 1 NURSİNG, BIRUNI UNIVERSITY, ISTANBUL; 2 NURSİNG, KOC UNIVERSTY, İSTANBUL, Türkiye

## Abstract

**Introduction:**

Phubbing with smartphones becoming an integral part of life and sociotelism behavior has emerged as an important academic concern (Chotpitayasunondh et al.,CHB,2016; 63, 9-18). Expectation of social support in social networks: It can be defined as “When the individual is emotionally intense, he/she shares about this situation and emotions and expects to be paid attention to, sincere and friendly approach from the people on social networking sites, and hopes that an environment of support and trust will be created with social networks by seeing that he/she is not alone with more emotional support.” (Uzakgiden et. al., JASR 2019;2 20-24)

In the nursing profession, in addition to using the internet for professional requirements, being a member of online social networking sites and spending time on these platforms are increasing.It is important to know the status of phubbing, which damages face-to-face communication understand its effect on patient-nurse communication and nurses communication within the team. Due to the intensity of work life in the nursing profession and the length of hours spent at work, the effort to meet the need for social support virtually may increase phubbing behavior.

**Objectives:**

The aim of this study was to examine the relationship among phubbing, online social support and trait anxiety, and the related factors in nurses.

**Methods:**

The population of the research consists of nurses who actively use their social media accounts. Nurses were accessed by snowball method on online Whatsapp groups, Instagram and Facebook platforms through a digital survey between May and November 2021. Data collected by using an individual descriptive characteristics form, generic scale of phubbing, online social support scale, trait anxiety inventory. Mann-Whitney U, Kruskal-Wallis H tests, t-test and pearson correlation coefficient was used for data analysis.

**Results:**

The phubbing scores of the nurses ranged from 15 to 98; the trait anxiety levels of the nurses were at a moderate level. It was observed that marital status, presence of WhatsApp groups with teammates, and being warned about the frequency of phone use from teammates was related to phubbingg (p<0.001). There was a positive moderate correlation between the phubbing score and the online social support score; a weak positive correlation was found between the general phubbing score and the trait anxiety score.

**Conclusions:**

It was determined that social support from social media significantly affected the phubbing behavior of nurses and that trait anxiety mediated this relationship. In future studies, researchers can conduct extensive research on the effect of sociotelism on nurse-patient communication and care. This study was also important in terms of making significant contributions to the literature, where there are only a limited number of studies on phubbing.

**Disclosure of Interest:**

None Declared

